# The Impact of Hospital Costing Methods on Cost-Effectiveness Analysis: A Case Study

**DOI:** 10.1007/s40273-018-0673-y

**Published:** 2018-05-22

**Authors:** José Leal, Stefania Manetti, James Buchanan

**Affiliations:** 1 Health Economics Research Centre, Nuffield Department of Population Health, University of Oxford, Old Road Campus, Headington, Oxford OX3 7LF UK; 2 Institute of Management, Scuola Superiore Sant’Anna, Pisa, Italy

## Abstract

**Background:**

Several methods exist to cost hospital contacts when estimating the cost effectiveness of a new intervention. However, the implications of choosing a particular approach remain unclear. We compare the use of the three main diagnosis-related group (DRG)-based national unit costs in England to determine whether choice of approach can impact on economic evaluation results.

**Methods:**

A cost-utility model was developed to compare secondary fracture prevention models of care for hip fracture patients, using data from large primary and hospital care administrative datasets in England. A healthcare and personal social services payer perspective was adopted, and utilities were informed by a meta-regression. Hospital resource use was valued using three DRG-based unit costs, and regression-based costing models were developed using data from 13,906 patients to inform the model health states.

**Results:**

Finished consultant episode (FCE)-level reference costs resulted in the highest costs on admission (£9075) and in the year of the fracture (£14,440). Relative to FCE-level costs, spell-level tariffs led to the lowest total hospital care costs per patient within 1 year of fracture (− £3691) compared with spell-level reference costs (− £2106). At a £20,000/quality-adjusted life-year threshold, using spell-level reference costs or spell-level tariffs, the introduction of a nurse-led fracture liaison service model of care was the cost-effective alternative. However, using FCE-level reference costs, usual care was the cost-effective option.

**Conclusions:**

Our results show that, conditional on the set of national unit costs adopted, the costs of hip fracture may vary considerably and different decisions may be reached regarding the introduction of new healthcare interventions.

**Electronic supplementary material:**

The online version of this article (10.1007/s40273-018-0673-y) contains supplementary material, which is available to authorized users.

## Key Points for Decision Makers

**Table:** 

There are several methods to cost hospital contacts when estimating the cost effectiveness of a new intervention; however, the implications of selecting a particular approach are unclear, potentially resulting in the over- or under-estimation of the costs associated with the intervention.
We found that the hospital costs associated with an exemplar condition (hip fracture) varied between £10,749 and £14,440 per fracture in the English National Health Service, depending on the set of unit costs used, impacting on both the lifetime costs of individuals and the total hospital costs of incident hip fracture in England.
Conditional on the set of national unit costs adopted, different policy decisions may be made regarding the introduction of new healthcare interventions. This may ultimately lead to suboptimal patient health outcomes, reducing population health.

## Introduction

There are several methods to cost hospital contacts when estimating the cost effectiveness of a new intervention. These can range from local micro-costing approaches to the use of diagnosis-related group (DRG)-based costs, which group patients according to their diagnosis and procedure codes as recorded in healthcare administrative records. In England, DRGs are called healthcare resource groups (HRGs) and the National Institute for Health and Care Excellence (NICE) recommends their use to cost hospital resource utilisation and inform economic evaluations [1]. However, analysts must choose between three main sources of HRG unit costs: (1) spell-level tariffs (commonly used to reimburse National Health Service [NHS] providers); (2) finished consultant episode (FCE)-level reference costs; and (3) spell-level reference costs.

A hospital spell, or hospital admission, comprises the total continuous stay of a patient using a hospital bed in the same hospital. During a hospital spell, a patient may receive medical care from one or more consultants. Time spent in the care of one consultant is called an FCE, and a hospital spell may contain one or more FCEs. Since 1997-98, reference cost data has been collected in England for all public-funded healthcare services (i.e. the NHS). Reference costs represent the cost of providing one unit of care in a given financial year and allow comparisons across hospital providers at the level of diagnosis, treatment and procedures [2, 3]. Reference costs reflect the direct, indirect and overhead costs associated with providing patient care and are collected from all NHS organisations at the FCE, spell and HRG level. The process is as follows: hospital-specific cost and activity data from a given financial year (e.g. 2014/2015) are collected in the following year (2015/2016) and analysed in the third year (2016/2017) to produce a set of national reference costs (2014/2015). In contrast, national tariffs are based on historical reference costs, filtered for services relevant to the tariff, inflated to tariff year prices, adjusted for unavoidable cost differences across region, and in some cases further adjusted downwards to incentivise the efficient delivery of medical care. Tariffs serve as national prices for healthcare services [4] and are a key source of acute provider income [3].

Tariffs for admitted patient activity are paid at spell level not FCE level as the Department of Health in England considers spells to be a more robust measure of hospital activity than FCEs. Spell-level costs were first collected in 2011–2012 alongside FCE-level costs [3]. These should ideally be based on patient-level costs or FCE mean costs if the former is not possible.

The analyst is therefore faced with three potential sources of unit costs for a given financial year to apply in an economic evaluation or cost-of-illness study. The implications of choosing a particular source of HRG-based unit costs when conducting costing studies and economic evaluations remain unclear. For example, the costs of a disease may be considerably underestimated or overestimated depending on which source of unit costs is used. Also, an intervention may be judged to be cost effective at a given willingness-to-pay threshold when a particular set of unit costs is used but not another.

We aim to address this gap in knowledge using hip fractures as a case study. Hip fractures are a major public health problem with significant patient morbidity and mortality and were estimated to cost £2–3 billion annually in health and social care costs in the UK [5, 6]. We estimated the cost variation of a hip fracture conditional on the source of HRG costs used and updated a cost-utility model developed to compare three secondary fracture prevention models of care for hip fracture patients [7]. The costs informing the cost-utility model were originally derived from the analysis of a large national hospital administrative dataset and we revisited these calculations using the three different HRG-based sets of unit costs for the financial year 2014–2015. For each source of HRG-based costs, we report the hospital costs of hip fracture as well as the absolute and incremental costs and incremental cost-effectiveness ratios (ICERs) associated with three models of secondary fracture prevention care.

## Methods

### Case Study

We developed a cohort transition model (Markov model) to estimate the lifetime costs, quality-adjusted life-years (QALYs) and cost effectiveness of three models of care for patients with a hip fracture admitted to an NHS hospital in England: (1) introduction of an orthogeriatrician (OG)-led service which focuses on achieving optimal recovery after hip fracture; (2) introduction of a nurse-led fracture liaison service (FLS) which focuses on secondary fracture prevention; and (3) standard post-hip fracture care (without the introduction and/or expansion of the OG and FLS models of care).

The cost-utility model is described in detail elsewhere [7]. Briefly, we developed the model in Microsoft Excel^®^ (Microsoft Corp., Redmond, WA, USA) to simulate the natural history, quality of life and costs of individuals with an index hip fracture across health states representing history of index hip fracture, second hip fracture, major non-hip fracture(s) (pelvic, spine, wrist, humerus and rib) requiring hospitalisation, living in patient’s own home or in a care home, and death (within 30 days post-hip fracture or within a year) (Electronic Supplementary Material, Figures A1 and A2). We used an iterative process to define the model involving discussions with clinical experts and epidemiologists, supplemented by a literature review of economic models in the area. An annual cycle length was adopted, the model was run until all individuals were dead (lifetime) and half-cycle correction was performed [8]. All costs from the original model were updated to 2014/2015 values and, together with outcomes, discounted at an annual rate of 3.5%. Hospital costs were updated after re-analysis of the data used to inform the original model (see Sect. 2.3).

Model inputs were derived from two main sources: Hospital Episode Statistics (HES) records and Clinical Practice Research Datalink (CPRD) records. The HES dataset comprised hospital records for 33,152 patients older than 60 years who had had an emergency hospital admission with a primary International Classification of Diseases, 10th revision (ICD-10) [24] diagnosis code for hip fracture (S72.0, S72.1, S72.2 and S72.9) between April 2003 and March 2013 for a representative region of the UK [9]. This dataset was used to estimate risk equations for the following events: time to second hip fracture, time to major non-hip fragility fracture requiring hospitalisation, discharge to care home (nursing or residential) after hip fracture, and time to death [7]. HES data between April 2009 and March 2013 were used to estimate the hospital hip fracture costs in the first year following fracture and the annual hospitalisation costs for each health state of the model (inpatient, outpatient, emergency and critical care costs as described in Sects. 2.2–2.5). This time period was chosen as adult critical care records have been available as a separate HES dataset since April 2008, allowing more precise costs to be estimated for each critical episode from this date onwards. The CPRD dataset comprised all primary care contacts, laboratory tests and prescribed drugs for 4063 patients registered in the CPRD GOLD database between 1 April 2003 and 31 March 2012, who had linked hospital records indicating a hip fracture. This dataset was used to estimate the annual primary care costs for each health state of the model.

Quality-of-life estimates were derived from a meta-regression, using a linear mixed-effects model, of 32 studies (21,085 patients) reporting preference-based quality of life [7]. All model input values and sources are described in detail in the Electronic Supplementary Material.

### Converting Hospital Data to Healthcare Resource Groups (HRGs)

HES data captures all hospital NHS patient care, as well as care for private patients treated in NHS hospitals and care delivered by treatment centres (including private providers) funded by the NHS. For each FCE, it contains anonymised patient administrative information (such as date of admission and discharge, admission method, age, sex and length of stay), diagnosis (ICD-10) and procedure codes (Office of Population Censuses and Surveys, 4th Revision [OPCS-4]). For the hospital cost analysis, we used HES data from April 2009 to March 2013 (4 years of data) comprising admitted patient care records, hospital outpatient activity (available from April 2003), adult critical care data (available from April 2008), and accident and emergency (A&E) attendances (available from April 2007). For each HES dataset, we derived two sets of 2014/2015 HRGs, one corresponding to the tariffs and one corresponding to the reference costs using specific Grouper software (*HRG4 2014*-*15 Payment Grouper* and *HRG4 + 2014*-*15 Reference Costs Grouper*) [10, 11]. The basis of reference costs in 2014/2015 was the HRG4 + , whereas the basis for payment tariffs for the same period was the HRG4. The number of HRGs increased from 1657 in the HRG4 to 2100 in the HRG4 + [12]. The Grouper software reads patient-level data at the FCE level to produce one HRG at FCE level and one HRG at spell level (which may differ). Furthermore, if there are additional high-cost elements related to the hospital episode and spell then additional HRGs are reported (called unbundled HRGs) so that these can be fully captured.

### Valuing HRGs

We matched the derived sets of HRGs at FCE and spell level to three sets of unit costs to convert hospital resource utilisation into 2014–2015 tariffs and costs:

- Spell-level tariffs 2014/2015 [13]
- FCE-level reference costs 2014/2015 [14]
- Spell-level reference costs 2014/2015 [14].

When the Grouper software produced an error code for the HRG at FCE or spell level (e.g. UZ01Z), we valued these HRGs using the weighted average of all HRGs (weights from FCE and spell activity as reported in the unit cost databases) by type of admission (elective, non-elective, day case, and regular day and night). If the length of stay exceeded the defined trimpoints for a given HRG, the cost of each additional bed day was added to the FCE-level costs or spell-level tariffs. Excess bed days were already included in the spell-level costs.

**Fig. 1:**
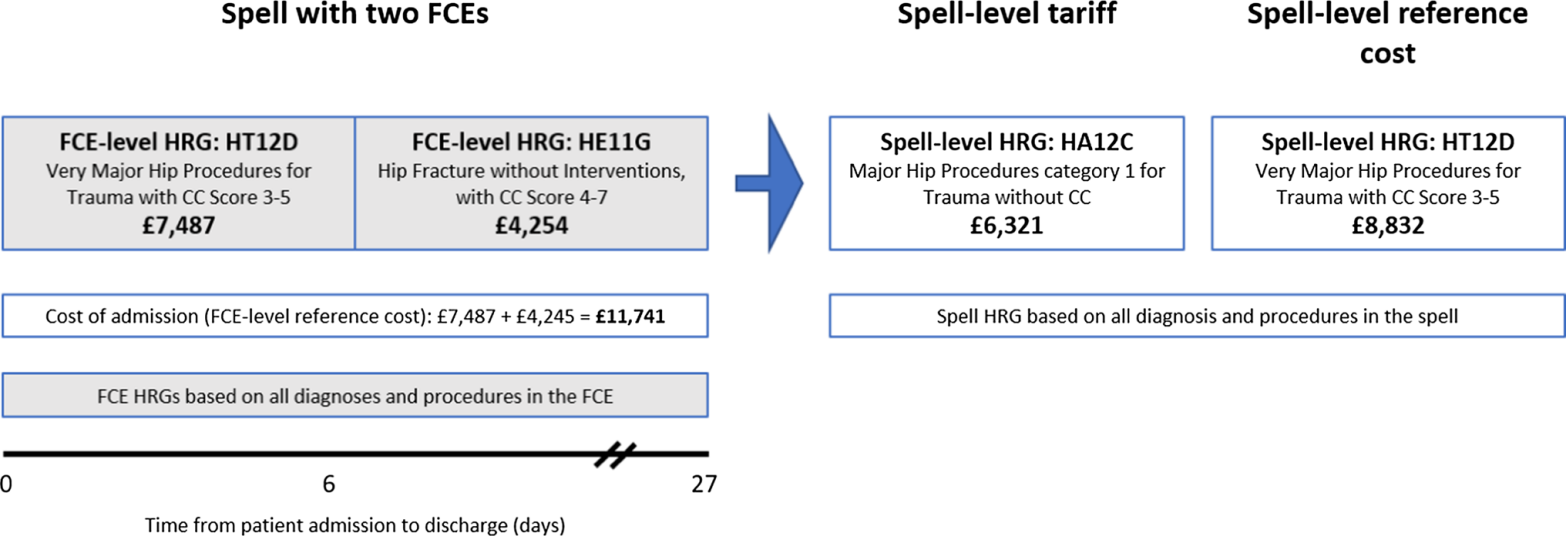
Valuing spell and finished consultant episode healthcare resource groups using reference costs and tariffs. This figure illustrates the potential impact of the decision to use one of the three sources of unit costs to value a single hospital stay. Using a hypothetical example of a patient being admitted with a hip fracture and having two finished consultant episodes during the hospital stay, the costs could vary between £6321 (using spell-level tariffs) and £11,741 (using finished consultant episode-level reference costs) based on the same patient and set of diagnosis, procedures and length of stay. In this example, spell-level tariffs for 2014/2015 were informed by HRG4, while reference costs for 2014/2015 were informed by HRG4+. *CC* complication or comorbidity, *FCE* finished consultant episode, *HRG* healthcare resource group

Spell-level reference costs are only collected for admitted patient care data (day cases, elective and non-elective inpatient stay) so we valued other types of hospital activity (critical care, outpatient visits/procedures, A&E attendance, unbundled HRGs) using the FCE-level reference costs dataset [14]. In the tariffs database of unit costs, some HRGs do not have national prices (e.g. critical care, dialysis for acute kidney injury, etc.) as they are subject to local prices to be contracted between commissioners and providers [13]. Furthermore, there are no data on the agreed local prices. Hence, we assumed that reference costs for the same period were proxies for the tariffs of the missing HRGs and cross-matched the HRG4 to HRG4+ codes, where required, using the patient-level diagnosis and procedure codes and the Reference Grouper software [10, 11]. This assumption reflects the calculation of tariffs where the reference costs of all NHS providers predates and informs the introduction of the tariff [3]. Figure 1 illustrates the potential impact of the decision to use one of the three sources of unit costs to value a single hospital stay.

### Statistical Analysis of Hospital Costs

Total hospital costs per patient were aggregated into annual amounts for the purposes of the analysis. We estimated the hospital costs of index fracture, hospital costs in the year of fracture and total annual hospital costs of incident hip fractures for the UK. The latter was estimated by multiplying the hospital costs in the year of fracture by the incidence of hip fracture in the UK (79,243 cases) [15]. The HES database was censored in 31 March 2013, and complete follow-up was not available for all cases. Adjusting for censoring using the methodology developed by Lin [16], we found the costs in the first 2 years of analysis to be very similar to a complete-case analysis [9]. Hence, we used complete cases to estimate the hospital costs in the first year following hip fracture as well as the annual costs associated with each health state.

Generalised linear models (GLMs) were used to predict annual hospital costs by health state. We estimated separate models for hospitalisation costs (inpatient and critical care) and non-hospitalisation costs (A&E and outpatient consultations). The following covariates were examined: sex, current age, age at hip fracture (first and second), living in a care home (nursing or residential), 30-day mortality following hip fracture, 1-year mortality following hip fracture, second hip fracture, major non-hip fracture requiring hospitalisation, history of second hip fracture, and history of major non-hip fracture. Covariates had to have a frequency of at least 100 patients to be considered for analysis, and were included in the final model if they were found to be statistically significant (*p* < 0.05). Model fit was assessed using Pregibon’s Link test and different family and link functions were compared using Akaike’s information criterion.

The distributions for the regression coefficients informing the models described above were obtained by bootstrapping the sample and re-estimating the regression models. This ensured that the correlation between coefficients and regressions was fully captured.

### Analysis

The impact of the three HRG-based sets of national unit costs was assessed in terms of absolute hospital costs and the relative cost effectiveness of the models of care. A hypothetical cohort of 1000 identical men was used to simulate the costs and QALYs of a representative patient with a hip fracture who is living in their own home before the fracture. The model was run three times using hospital costs based on the different HRG-based sets of costs. A model of care was deemed to be cost effective if the ICER was below £20,000 per QALY gained [1] The ICER was estimated by dividing the difference in mean costs by the difference in mean effects (life-years and QALYs) for a given model of care compared with its next best alternative. The internal validity of the model was checked using sensitivity analysis (extreme values) and by comparing the model outputs with the data used to build the model. Parameter uncertainty was evaluated using probabilistic sensitivity analysis and quantified using a cost-effectiveness acceptability curve (CEAC) [17].

## Results

### Using the Patient Sample to Estimate Hospital Costs

Between 1 April 2009 and 31 March 2013, 13,906 patients were identified as having had a hip fracture (Table 1). The mean age of the sample was 83 years and 73% were female. Most patients were of white ethnicity. The average follow-up of the cohort was 1.4 years. For cases with complete follow-up in the first year, the mean length of stay was 20.1 days in the index admission and 35.7 days in the year of the fracture.

**Table 1:** Characteristics of individuals with hip fracture between 2009 and 2013

Variable	Value
Number of individuals with index hip fracture	13,906
Age (years) [mean (SD)]	83.0 (8.4)
Males [*n* (%)]	3814 (27)
White ethnicity [*n* (%)]^a^	13,402 (99)
Charlson comorbidity index score [mean (SD)]	1.6 (0.1)
Top 3 complications recorded in previous hospitalisations [*n* (%)]	
Dementia	3021 (22)
Pulmonary disease	2432 (17)
Diabetes mellitus	1907 (14)
Source of admission at index fracture [*n* (%)]	
Own home	11,696 (84)
Nursing/residential/temporary accommodation	1667 (12)
Other	543 (4)
Follow-up time (years) [mean (SD)]	1.4 (1.2)
Mortality [*n* (%)]	
Within 30 days^b^	1103 (8)
Within 1 year^c^	3580 (32)
Discharge destination following index fracture admission [*n* (%)]	
Own home	7332 (53)
Nursing/residential/temporary accommodation	2682 (19)
National Health Service hospital	2545 (18)
Other	1347 (10)
Length of stay within 1 year of fracture^c^ [mean (SD)]	
Initial hospitalisation	20.1 (18.5)
Total	35.7 (36.8)

*SD* standard deviation

^a^346 missing

^b^Cases with complete follow-up during the 30 days following index fracture (*n* = 13,743)

^c^Cases with complete follow-up, including those who died in that year (*n* = 11,184)

### Absolute Costs of Hip Fracture

Table 2 reports the mean hospital costs associated with hip fracture, estimated using the three different sets of national unit costs. Use of the FCE-level reference costs database resulted in the highest costs at the index admission (£9075) and in the year of the fracture (£14,440). Relative to FCE-level costs, use of spell-level tariffs led to the lowest total hospital care costs per patient within 1 year of fracture (a difference of –£3691, 95% confidence interval [CI] £3597 to − £3785) compared with spell-level reference costs (− £2106, 95% CI − £1987 to − £2226). Across all HRG-based sets of national unit costs, 96% of costs in the year of the fracture were due to inpatient stay and critical care.

**Table 2:** Hospital costs in the year of hip fracture, by source of unit costs

Costs	Mean costs (£) (95% confidence interval)^a^		
	FCE-level reference costs	Spell-level reference costs	Spell-level tariffs
Initial inpatient care costs (index admission to discharge)	9075 (9035–9197)	8145 (8097–8193)	6689 (6635–6744)
Inpatient care costs within 1 year of fracture	13,866 (13,676–14,055)	11,759 (11,627–11,892)	10,263 (10,126–10,400)
A&E and outpatient care costs within 1 year of fracture	575 (561–588)	575 (561–588)	486 (472–500)
Total hospital care costs within 1 year of fracture	14,440 (14,248–14,633)	12,334 (12,197–12,471)	10,749 (10,609–10,889)

*A&E* accident and emergency, *FCE* finished consultant episode

^a^Individuals with at least 1 year of follow-up, alive or dead (*n* = 11,184)

Table 3 reports the index hospital costs conditional on the number of FCEs. As the number of FCEs in a spell increases, the difference between using FCE-level reference costs and the other two national databases increases. For index admissions with only one FCE, the highest costs resulted from using the spell-level reference costs followed by FCE-level reference costs (− £694; *p* < 0.001) and spell-level tariffs (− £1738; *p* < 0.001). In contrast, index admissions with two FCEs were valued £2850 (*p* < 0.001) and £3922 (*p* < 0.001) higher using FCE-level reference costs than using spell-level reference costs and spell-level tariffs, respectively. Index admissions with three or more FCEs were £7308 (*p* < 0.001) and £7778 (*p* < 0.001) higher if valued using FCE-level reference costs than using spell-level reference costs and spell-level tariffs, respectively. Using spell-level tariffs resulted in the lowest costs of index admissions. Table A15 in the Electronic Supplementary Material reports the top five HRGs in our dataset for index admissions with one FCE according to the three sets of unit costs.

**Table 3:** Hospital costs in the index admission, by source of unit costs and number of finished consultant episodes per admission

Index admission to discharge	Frequency (%)	Mean costs (£) (95% confidence interval)		
		FCE-level reference costs	Spell-level reference costs	Spell-level tariffs
One FCE	9257 (67)	7375 (7317–7432)	8069 (8021–8116)	6331 (6274–6387)
Two FCEs	3279 (24)	10,837 (10,703–10,970)	7986 (7878–8095)	6915 (6800–7030)
Three or more FCEs	1370 (9)	16,352 (15,917–16,786)	9043 (8785–9301)	8573 (8297–8850)

*FCE* finished consultant episode

**Fig. 2:**
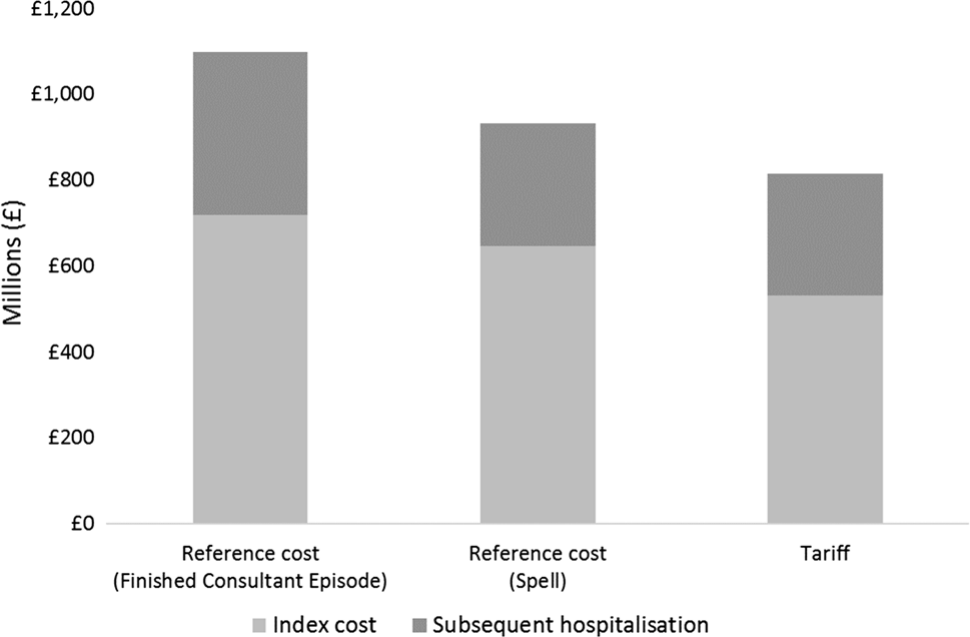
Total annual hospital costs associated with incident hip fracture in the UK conditional on source of unit costs

The total annual hospital costs associated with all incident hip fractures in the UK were estimated to vary between £813 million (spell-level tariffs) and £1099 million (FCE-level reference cost) per year depending on the source of unit costs used (Fig. 2).

### Statistical Analysis of Hospital Costs

Between 2009 and 2013, there were 19,644 patient-years of data available in the HES dataset to estimate the hospital cost equations. Tables A5–A14 in the Electronic Supplementary Material report the regression models for hospitalisation (admitted patient care and critical care) and non-hospitalisation costs (A&E and outpatient) based on the three sets of national unit costs. Overall, the direction of the coefficients was consistent across the three sets of unit costs. However, the magnitude and statistical significance of the covariates varied depending on the unit costs used.

For example, hospitalisation costs in year of second hip fracture were significantly associated with death within 30 days of hip fracture using FCE-level reference costs and spell-level tariffs but not with spell-level reference costs (Electronic Supplementary Material Table A6). Conditional on hospitalisation and death, living in a care home was significantly associated with higher hospitalisation costs if HRGs were valued using reference costs at FCE-level or national tariffs, but the association was no longer significant using reference costs at spell level (Electronic Supplementary Material Table A9).

### Representative Male Patient

The average age of a male patient with a hip fracture, not living in a care home, was 81.4 years with a Charlson co-morbidity index (CCI) score of 1.9. Table 4 reports the total QALYs and costs (healthcare and social care) associated with usual care of a representative  male patient. Using FCE-level reference costs, over the lifetime of the patient, we would expect usual care to cost £39,906 and result in 2.57 life-years (discounted). Relative to FCE-level reference costs, the mean discounted total costs were estimated to be £4057 and £5999 lower when based on spell-level reference costs and spell-level tariffs, respectively. Care home costs accounted for 32–35% of total discounted costs, depending on the set of unit costs used.

**Table 4:** Lifetime costs and quality-adjusted life-years, by source of unit costs^a^

	FCE-level reference costs (£)	Spell-level reference costs (£)	Spell-level tariffs (£)	Life-years	QALYs
Usual care	39,906 (38,449–41,469)	35,848 (34,588–37,728)	33,907 (32,606–35,352)	2.57 (2.46–2.68)	1.58 (1.39–1.77)
FLS vs. usual care	2058 (1322–2765)	1896 (1221–2503)	1921 (1254–2544)	0.16 (0.10–0.22)	0.10 (0.06–0.14)
Orthogeriatrician vs. FLS	678 (–220 to 1500)	632 (–162 to 1460)	646 (–196 to 1515)	0.04 (–0.03 to 0.12)	0.03 (–0.02 to 0.08)

Data are given as mean (95% confidence interval)

*FCE* finished consultant episode, *FLS* nurse-led fracture liaison services, *QALYs* quality-adjusted life-years

^a^Discounted at 3.5%

For our male cohort, the most effective model of care was the introduction of an OG, followed by the introduction of an FLS and then usual care. On average, when compared with usual care, FLS and OG-led models of care resulted in an additional 0.10 and 0.13 QALYs gained (discounted), respectively. At a £20,000/QALY threshold, using spell-level reference costs or spell-level tariffs, the introduction of a nurse-led FLS model of care was the cost-effective alternative. The probability that this was the cost-effective option was estimated at 53% (Table 5 and Electronic Supplementary Material Figure A3). However, using FCE-level reference costs, usual care was the cost-effective option at the £20,000/QALY threshold.

**Table 5:** Cost-effectiveness analysis results, by source of unit cost

Comparison	Measure	FCE-level reference costs	Spell-level reference costs	Spell-level tariffs
FLS vs. usual care	ICER	£20,605	£18,982	£19,228
	Probability of being most cost-effective option^a^	29%	53%	53%
Orthogeriatrician vs. FLS	ICER	£23,958	£22,359	£22,843
	Probability of being most cost-effective option^a^	8%	27%	21%

*FCE* finished consultant episode, *FLS* nurse-led fracture liaison services, *ICER* incremental cost-effectiveness ratio

^a^Intervention judged to be cost effective if ICER was below £20,000 per quality-adjusted life-year

## Discussion

Our study illustrates the implications of choosing a particular source of HRG-based national unit costs when conducting costing studies and economic evaluations. To demonstrate this, we used a large dataset of hospital administrative records to estimate the costs of hip fracture and the inputs of a decision model based on three national unit cost datasets. We found that the hospital costs of hip fracture in the year of the event varied between £10,749 and £14,440 per fracture depending on the set of unit costs used. These differences impacted on the lifetime costs of individuals as well as the total hospital costs of incident hip fracture in the UK, which varied between £813 million and £1099 million per year. In addition to the impact on absolute costs, the methodological uncertainty of the cost models was considerable. Some of the predictors of costs that were significant with one dataset of costs were no longer significant using another dataset of costs.

In the reference case for technology appraisal [1], NICE recognises HRGs as a valuable source of hospital resource use and recommends the use of national unit costs collected in the form of reference costs. However, the reference case does not explicitly exclude the use of national tariffs nor sets out the type of reference cost database to use. In our case study and at a threshold of £20,000/QALY, we found that there was a notable impact on the recommendation of which intervention to implement conditional on the source of unit costs adopted. Using reference costs at spell level and tariffs resulted in FLS-led services being the most cost-effective option, whereas usual care was found to be the most cost-effective option using reference costs at FCE-level. The impact of the source of unit costs on incremental costs and incremental cost effectiveness is smaller than on absolute costs, with the ICER varying from £18,982/QALY to £20,605/QALY for FLS compared with usual care. However, in specific cases where the ICER is close to the maximum willingness-to-pay value, this is likely to impact on the suggested adoption decision. Policy makers, researchers and analysts should therefore be aware of this issue.

Reference costs were initially collected to facilitate comparisons between hospitals. However, their use has been further expanded to inform local payments, academic research and national decisions concerning the implementation of novel interventions based on economic evaluations of novel interventions. Hence, every year the Department of Health in England collects data from NHS providers and commissioners on all running costs of providing services at FCE level and, recently, at spell level for admitted patient care services. In 2014–2015, FCE-based reference costs captured £61 billion of NHS expenditure (55% of total expenditure), of which £25 billion concerned admitted patient care. Spell-based reference costs captured £25 billion in the same period [2]. Our results highlight concerns about the quality of reference cost data [18, 19]. The reason for the significant differences in costs of hospital admissions using FCE-based and spell-based reference costs is unclear. For example, for index hospital admissions comprising two or more FCEs, we would expect hospital admission costs to be lower when based on spell-level unit costs due to potential savings on hospital entry or consultant transfer costs. However, we did not expect these differences to be as high as those observed (£2850 and £7308 for two or more FCEs, respectively), raising questions about the spell-based HRG allocation algorithms and the accuracy of the validation checks of spell and FCE costs submitted by each hospital provider. Furthermore, for index admissions with a single FCE, using spell-based unit costs resulted in higher hospital admission costs than using FCE-based unit costs. In these cases, the estimated costs using spell-based unit costs were consistently higher across all HRGs than using FCE-based unit costs, perhaps reflecting an FCE to spell ratio > 1 for index admissions. We also found that the spell-level and FCE-level HRGs did not always agree for the same patient. There is clearly a need for hospital providers to record and report cost data more accurately as well as for more transparently calculated costs and tariffs.

This is the first study to evaluate the implications of choosing different HRG-based national unit costs when conducting costing studies and to then use the resulting cost estimates within economic evaluations. A key strength of our study is that we were able to utilise a large linked dataset of hospital and clinical practice records that allowed us to ensure that observed differences were precisely estimated. Our work builds on two earlier related studies. The first study, by Geue et al. [20], compared five methods of costing HES using data from Scotland on acute hospital admissions, applying HRG version 3.5 Grouper software. The different approaches were based on HRG codes, used information on per diem costs, or derived specialty specific costs using information on length of stay. Substantial differences in cost estimates were observed, with approaches tied to length of stay yielding higher costs. The study concludes by recommending the use of a specific HRG costing method.

The second study, by Thorn et al. [21], evaluated the inpatient costs of 292 men with prostate cancer using two approaches: HES data combined with NHS reference costs; and costs derived from a review of medical records. Again, HRG version 3.5 Grouper software was used. The key finding was that the costs estimated using the HES approach were 8% lower than those estimated via medical record review. However, this was not a significant difference.

Our work moves this literature forward by using linked data on a variety of hospital activities (not just acute care) over a long time period for a large sample to investigate the impact of choice of costing approach on economic evaluation results. For this reason, we believe that our findings are broadly generalisable to other costing studies and economic evaluations in an English NHS context. Several limitations of our study should, however, be noted. It would have been useful to contrast the results against those obtained using a micro-costing approach. However, given the scale of the exercise and the available dataset, this was not judged to be appropriate or feasible. Furthermore, our aim was to replicate what is most likely to occur in practice when a researcher needs to cost a disease or perform an economic evaluation. Spell-based reference costs only concern admitted patient care and, as a result, non-admitted patient care (A&E, outpatient visits and procedures) were valued using the FCE-based reference costs. Spell-based tariffs were missing for some HRGs as they are subject to local prices to be contracted between commissioners and providers [13]; we used as a proxy the FCE-based reference costs for the same year. These assumptions likely reduced the estimated differences in costs in the three analyses. However, the impact of this effect is likely to be small as the contribution of the non-admitted patient care costs to total costs is small and the proportion of hospitalisation data with missing spell-based tariffs was limited. A final point to note is that 2014/2015 reference costs were based on HRG4+, whereas in the same period payment tariffs were based on HRG4 (which contained fewer HRGs). However, the impact of this change on our results is unclear.

We hope that the results of our study will be informative for analysts who need to select a set of HRG-based unit costs for a future costing study or economic evaluation. The appropriate set of unit costs to use will vary depending on the analytical perspective that is adopted; hence, it is not possible to make an overall recommendation regarding this analytical decision. If the broad aim of a study is to inform resource allocation, analysts may prefer to apply reference costs as these are likely to be the best proxy for opportunity costs. Alternatively, if a study is being conducted from the perspective of a hospital provider, tariffs may be the more appropriate choice. It should also be noted that the Department of Health favour the use of FCE-based reference costs rather than spell costs [14]. Health technology assessment agencies in other countries, such as Canada and Australia, have been more ambiguous about the appropriate approach to use in different circumstances [22, 23]. Finally, regardless of the approach selected in the base-case analysis of a study, if the ICERs generated in an economic evaluation fall close to the threshold of the relevant decision maker, and/or considerable uncertainty is observed in the CEACs, it would be good practice for analysts to conduct sensitivity analyses in which alternative sets of unit costs are applied.

## Conclusion

As the availability of large administrative records increases it becomes important to ensure that such data are analysed appropriately and the methods used are documented fully. Our results show that, conditional on the set of national unit costs adopted, the cost of diseases may vary considerably and different policy decisions may be made regarding the introduction of new healthcare interventions. The variability in cost estimates may impair healthcare planning and any misallocation of scarce healthcare resources may ultimately lead to suboptimal patient health outcomes, reducing population health.

### **Data Availability Statement**

The data underlying the analyses in this paper cannot be made publicly available as this is not permitted by the data supplier, NHS Digital.

## Electronic supplementary material

Below is the link to the electronic supplementary material.

Supplementary material 1 (DOCX 228 kb)

## References

[1] National Institute for Health and Care Excellence. Guide to the methods of technology appraisal 2013. https://www.nice.org.uk/process/pmg9/2013. Accessed 28 Jan 2018. 27905712

[2] Department of Health. Reference costs 2015-16. https://www.gov.uk/government/uploads/system/uploads/attachment_data/file/577083/Reference_Costs_2015-16.pdf. Accessed 28 Jan 2018.

[3] Department of Health. A simple guide to Payment by Results. https://www.gov.uk/government/uploads/system/uploads/attachment_data/file/213150/PbR-Simple-Guide-FINAL.pdf. Accessed 28 Jan 2018.

[4] Monitor. NHS England. 2016/17 National Tariff Payment System. https://www.gov.uk/government/uploads/system/uploads/attachment_data/file/509697/2016-17_National_Tariff_Payment_System.pdf. Accessed 28 Jan 2018.

[5] Hernlund E, Svedbom A, Ivergard M, Compston J, Cooper C, Stenmark J, et al. Osteoporosis in the European Union: medical management, epidemiology and economic burden. A report prepared in collaboration with the International Osteoporosis Foundation (IOF) and the European Federation of Pharmaceutical Industry Associations (EFPIA). Arch Osteoporos. 2013;8(1–2):136. 10.1007/s11657-013-0136-1 PMC3880487 24113837

[6] Burge RT, Worley D, Johansen A, Bhattacharyya S, Bose U (2001). The cost of osteoporotic fractures in the UK: projections for 2000–2020. J Med Econ..

[7] Leal J, Gray AM, Hawley S, Prieto-Alhambra D, Delmestri A, Arden NK (2017). Cost-effectiveness of orthogeriatric and fracture liaison service models of care for hip fracture patients: a population-based study. J Bone Miner Res.

[8] Barendregt JJ (2014). The life table method of half cycle correction: getting it right. Med Decis Making.

[9] Leal J, Gray AM, Prieto-Alhambra D, Arden NK, Cooper C, Javaid MK (2016). Impact of hip fracture on hospital care costs: a population-based study. Osteoporos Int.

[10] NHS Digital. HRG4 2014-15 Payment Grouper. http://webarchive.nationalarchives.gov.uk/20171011193155. Accessed 14 May 2018.

[11] NHS Digital. HRG4 + 2014-15 Reference Costs Grouper. http://webarchive.nationalarchives.gov.uk/20171011071739/http://content.digital.nhs.uk/article/6226/HRG4-201415-Reference-Cost-Grouper. Accessed 28 Jan 2018.

[12] NHS Digital. HRG4+ summary of changes. http://webarchive.nationalarchives.gov.uk/20180328130852tf_/http://content.digital.nhs.uk/media/11601/Summaryof-Changes/pdf/HRG4__RC12-13_Summary_of_Changes_v1.0.pdf/. Accessed 28 Jan 2018.

[13] Monitor. NHS England. National tariff payment system 2014/15. https://www.gov.uk/government/publications/national-tariff-payment-system-2014-to-2015. Accessed 28 Jan 2018.

[14] Department of Health. NHS reference costs 2014 to 2015. https://www.gov.uk/government/publications/nhs-reference-costs-2014-to-2015. Accessed 28 Jan 2018.

[15] Hernlund E, Svedbom A, Ivergård M, Compston J, Cooper C, Stenmark J (2013). Osteoporosis in the European Union: medical management, epidemiology and economic burden: a report prepared in collaboration with the International Osteoporosis Foundation (IOF) and the European Federation of Pharmaceutical Industry Associations (EFPIA). Arch Osteoporos..

[16] Lin DY (2000). Linear regression analysis of censored medical costs. Biostatistics..

[17] Fenwick E, Claxton K, Sculpher M (2001). Representing uncertainty: the role of cost-effectiveness acceptability curves. Health Econ.

[18] Delloitte LLP. Reference cost data quality. A final report for Monitor. 2014. https://www.gov.uk/government/uploads/system/uploads/attachment_data/file/317571/Supporting_document_B_-_Deloitte_Reference_Cost_Data_Quality_for_publication8e14.pdf. Accessed 28 Jan 2018.

[19] Monitor. Reference cost assurance programme: findings from the 2014/15 audit. 2015. https://www.gov.uk/government/uploads/system/uploads/attachment_data/file/466112/Reference_cost_audit_report_final_v2.pdf. Accessed 28 Jan 2018.

[20] Geue C, Lewsey J, Lorgelly P, Govan L, Hart C, Briggs A (2012). Spoilt for choice: implications of using alternative methods of costing hospital episode statistics. Health Econ.

[21] Thorn JC, Turner EL, Hounsome L, Walsh E, Down L, Verne J (2016). Validating the use of Hospital Episode Statistics data and comparison of costing methodologies for economic evaluation: an end-of-life case study from the Cluster randomised triAl of PSA testing for Prostate cancer (CAP). BMJ Open.

[22] Canadian Agency for Drugs and Technologies in Health. guidance document for the costing of health care resources in the Canadian setting: hospital services. https://www.cadth.ca/dv/guidance-document-costing-health-care-resources-canadian-setting. Accessed 3 May 2018.

[23] The Pharmaceuticals Benefits Scheme. Manual of resource items and their associated unit costs: hospital services. http://www.pbs.gov.au/info/industry/useful-resources/manual-pages/6-hospital-services. Accessed 3 May 2018.

[24] International classification of diseases (1992). 10th revision.

