# Correction to: The Impact of Hospital Costing Methods on Cost-Effectiveness Analysis: A Case Study

**DOI:** 10.1007/s40273-018-00764-3

**Published:** 2019-03-05

**Authors:** José Leal, Stefania Manetti, James Buchanan

**Affiliations:** 1 Health Economics Research Centre, Nuffield Department of Population Health, University of Oxford, Old Road Campus, Headington, Oxford OX3 7LF UK; 2 Institute of Management, Scuola Superiore Sant’Anna, Pisa, Italy

## Correction to: PharmacoEconomics (2018) 36:1263–1272 10.1007/s40273-018-0673-y

The Open Access license, which previously read:

**Open Access** This article is distributed under the terms of the Creative Commons Attribution-NonCommercial 4.0 International License (http://creativecommons.org/licenses/by-nc/4.0/), which permits any noncommercial use, distribution,and reproduction in any medium, provided you give appropriate credit to the original author(s) and the source, provide a link to the Creative Commons license, and indicate if changes were made.

Should read:

**Open Access** This article is distributed under the terms of the Creative Commons Attribution 4.0 International License (http://creativecommons.org/licenses/by/4.0/), which permits unrestricted use, distribution, and reproduction in any medium, provided you give appropriate credit to the original author(s) and the source, provide a link to the Creative Commons license, and indicate if changes were made.

The original article has been corrected.

