# Supplementary material for: The Impact of Hospital Costing Methods on Cost-Effectiveness Analysis: A Case Study
Source: Pharmacoeconomics. 2018 May 22;36(10):1263–72. doi: 10.1007/s40273-018-0673-y (PMC6132447; doi:10.1007/s40273-018-0673-y)
Supplement: Supplementary file 1 — Supplementary material 1 (DOCX 228 kb) [file 40273_2018_673_MOESM1_ESM.docx]

**The impact of hospital costing methods on cost-effectiveness analysis**

**Supplementary material**

Jose Leal^1^, Stefania Manetti^2^, James Buchanan^1*^

^1^ Health Economics Research Centre, Nuffield Department of Population Health, University of Oxford, Old Road Campus, Headington, Oxford, UK

^2^ Institute of Management, Scuola Superiore Sant'Anna, Pisa, Italy

***** correspondence to: James Buchanan, Health Economics Research Centre, Nuffield Department of Population Health, University of Oxford, Old Road Campus, Headington, Oxford, OX3 7LF. Tel: (01865 289262). Email: [james.buchanan@dph.ox.ac.uk](mailto:james.buchanan@dph.ox.ac.uk)

**SECTION 1: FURTHER DETAILS ABOUT THE DECISION MODEL**

The decision model is described in more detail in Leal *et al.*[1] In this appendix we provide further information about the model structure, parameters, risk equations, cost equations and the probabilistic sensitivity analysis.

Model structure

In the cycles subsequent to index hip fracture, patients were classified into four health states (history of index hip fracture, history of non-hip fractures requiring hospitalisation, history of second hip fracture and history of second hip fracture and other non-hip fracture) conditional on living in their own home or a care home. Patients were allowed to continue progressing (e.g. second hip fracture, other major fractures, death, no events) if they had not yet reached an absorbing state (death and history of second hip fracture and other major fragility fracture). **Figures A1 and A2** describe the model structure for patients who are discharged to a care home, and patients who are discharged to their own home.

**Figure A1: Model structure and allowed transitions in the years following the index hip fracture for patients discharged to a care home**

**Figure A2: Model structure and allowed transitions in the years following the index hip fracture for patients discharged to their own home**

Model inputs

**Table A1** reports the inputs informing the decision model. The predictions from the primary care regression models were updated to 2014/15 prices using the pay and price index.[2] The distributions for the regression coefficients informing the models described in **Tables A2-A4** were obtained by bootstrapping the sample and re-estimating the regression models. This ensured that the correlation between coefficients was fully captured. The distributions used for the remaining parameters were selected according to recommended practice.[3] Relative effectiveness measures (i.e. hazard ratios) were modelled using a lognormal distribution. Parameters concerning proportions/probabilities were modelled using beta distributions.

**Table A1: Model parameters**

| **Parameter** | **Mean (95% confidence interval)** | **Source** |
| --- | --- | --- |
| Relative effectiveness (hazard ratio) |  |  |
| Introducing OG relative to usual care |  |  |
| 30-day mortality following index hip fracture | 0.73 (0.65-0.82) | Hawley 2016 [4] |
| 1-year mortality following index hip fracture | 0.81 (0.75-0.87) | Hawley 2016 [4] |
| 2-year risk of developing second hip fracture | 0.95 (0.79-1.15) | Hawley 2016 [4] |
| Introducing FLS relative to usual care |  |  |
| 30-day mortality following index hip fracture | 0.80 (0.71-0.91) | Hawley 2016 [4] |
| 1-year mortality following index hip fracture | 0.84 (0.77-0.93) | Hawley 2016 [4] |
| 2-year risk of developing second hip fracture | 1.03 (0.85-1.26) | Hawley 2016 [4] |
| Discharge to care home after index hip fracture (previously not in care home) | Table A2 | Leal 2017 [1] |
| Discharge to care home after second hip fracture (previously not in care home) | Table A2 | Leal 2017 [1] |
| Risk of second hip fracture | Table A3 | Leal 2017 [1] |
| Risk of major non-hip fracture requiring hospitalisation | Table A3 | Leal 2017 [1] |
| 30-day mortality after index hip fracture | Table A3 | Leal 2017 [1] |
| 30-day mortality after second hip fracture | Table A3 | Leal 2017 [1] |
| All-cause mortality post 30 days of fracture | Table A3 | Leal 2017 [1] |
| Intervention cost ^a^ |  |  |
| Fracture liaison nurse (Grade 7) per hip fracture patient | £203 | Leal 2017 [1] |
| Orthogeriatrician (Consultant) | £418 | Leal 2017 [1] |
| Proportion discharged to a care home that go to a nursing home | 0.48 (0.47-0.49) | Leal 2017 [1] |
| Primary care costs in year of index fracture | Table A4 | Leal 2017 [1] |
| Primary care costs in years after index fracture | Table A4 | Leal 2017 [1] |
| Hospital costs in year of index hip fracture | Tables A5 and A10 | This study |
| Hospital costs in year of second hip fracture | Tables A6 and A11 | This study |
| Hospital costs in years following fracture | Tables A7 and A12 | This study |
| Hospital costs if non-hip fracture occurs | Tables A8 and A13 | This study |
| Hospital costs if death occurs | Tables A9 and A14 | This study |
| Cost of institutionalisation (per year) ^b^ |  |  |
| Nursing home | £40,040 | Curtis 2016 [2] |
| Residential home | £32,656 | Curtis 2016 [2] |
| Utility of hip fracture patients |  |  |
| Within 1 month of index fracture | 0.46 (0.38-0.55) | Leal 2017 [1] |
| At 12 months | 0.53 (0.47-0.61) | Leal 2017 [1] |
| At 24 months and after | 0.66 (0.60-0.74) | Leal 2017 [1] |
| Discount rate for costs and outcomes | 3.5% | HM Treasury [5] |

^a^ includes salary (2014-15), salary oncosts, qualification costs, management and non-staff overheads and capital overheads. ^b^ Nursing home at £40,040 per year (£770 per week for 52 weeks) and residential home at £32,656 per year (£628 per week for 52 weeks). HES = Hospital Episode Statistics database.

**Table A2: Risk equations estimating the probability of admission to a care home (HES data April 2003 - March 2013)**

| **Event** | **Care home after first hip fracture** | | **Care home after second hip fracture** | |
| --- | --- | --- | --- | --- |
| Patients | 24,879 | | 1,599 | |
| Number of events | 4,869 | | 278 | |
| Functional form | Logistic | | Logistic | |
| **Parameters** | **Mean** | **SE** | **Mean** | **SE** |
| Constant | -10.490 | 1.398 | -4.120 | 0.777 |
| Female | 0.152 | 0.040 | - | - |
| Age at first hip fracture | 0.173 | 0.034 | - | - |
| (Age at first hip fracture)^2 | -0.0008 | 0.0002 | - | - |
| Age at second hip fracture | - | - | 0.030 | 0.009 |
| CCI score at first hip fracture | 0.042 | 0.011 | - | - |
| p>X^2^ | <0.001 | | <0.001 | |

CCI score = Charlson co-morbidity index; SE = standard error.

**Table A3: Risk equations estimating the probability of events and all-cause mortality (HES data April 2003 - March 2013)**

| **Event** | **Second hip fracture** | | **Major non-hip fracture** | | **30-day all-cause mortality after first hip fracture** | | **30-day all-cause mortality after second hip fracture** | | **All-cause mortality post 30 days** | |
| --- | --- | --- | --- | --- | --- | --- | --- | --- | --- | --- |
| Patients | 29,888 | | 29,888 | | 32,989 | | 2,197 | | 29,888 | |
| Number of events | 2,206 | | 1,464 | | 3,101 | | 173 | | 13,008 | |
| Functional form | Weibull | | Weibull | | Logistic | | Logistic | | Gompertz | |
| **Parameters** | **Mean** | **SE** | **Mean** | **SE** | **Mean** | **SE** | **Mean** | **SE** | **Mean** | **SE** |
| Constant | -6.951 | 0.244 | -6.867 | 0.298 | -8.705 | 0.242 | -6.264 | 1.014 | -7.471 | 0.143 |
| Ρ | 1.099 | 0.018 | 1.259 | 0.024 | - | - | - | - | - | - |
| Γ | - | - | - | - | - | - | - | - | 0.012 | 0.005 |
| Female | 0.117 | 0.055 | 0.481 | 0.077 | -0.505 | 0.043 | -0.623 | 0.186 | -0.436 | 0.020 |
| Age at first hip fracture | 0.042 | 0.003 | 0.028 | 0.004 | 0.075 | 0.003 | - | - | 0.072 | 0.002 |
| Age at second hip fracture | - | - | - | - | - | - | 0.048 | 0.012 | - | - |
| Care home | - | - | 0.451 | 0.055 | 0.236 | 0.057 | 0.360 | 0.170 | 2.092 | 0.219 |
| CCI score at first hip fracture | - | - | - | - | 0.269 | 0.010 | - | - | 0.655 | 0.062 |
| Major non-hip fracture | 0.371 | 0.117 | - | - | - | - | - | - | - | - |
| Second hip fracture | - | - | 0.377 | 0.121 | - | - | - | - | - | - |
| History of non-hip fracture | - | - | - | - | - | - | - | - | 0.152 | 0.053 |
| History of second hip fracture | - | - | 0.286 | 0.113 | - | - | - | - | 0.246 | 0.044 |
| Age X care home | - | - | - | - | - | - | - | - | -0.020 | 0.003 |
| Age X CCI score at first hip fracture | - | - | - | - | - | - | - | - | -0.006 | 0.001 |
| p>X^2^ | <0.001 | | <0.001 | | <0.001 | | <0.001 | | <0.001 | |

CCI score: Charlson co-morbidity index at hospital admission for primary hip fracture (up to 3 years before); SE = standard error; Age X care home = interaction term for age at primary hip fracture and living in a care home; Age X CCI = interaction term for age and CCI score at primary hip fracture.

**Table A4:** **Primary care cost equations**

|  | **Year of first hip fracture** | | **Subsequent years** | |
| --- | --- | --- | --- | --- |
| Number of observations | 3,910 | | 7,373 | |
| Number of patients | 3,910 | | 2,568 | |
| Distributional form | Gamma | | Gamma | |
| Link function | Identity | | Identity | |
| **Parameters** | **Mean** | **SE** | **Mean** | **SE** |
| Constant | 1,251 | 39 | 1,161 | 40 |
| Death within 30 days | -1,197 | 52 | - | - |
| Death within year | -689 | 36 | -437 | 52 |
| Living in care home | 126 | 39 | - | - |
| Major non-hip fracture | - | - | 502 | 264 |

SE = standard error.

**SECTION 2: ADDITIONAL RESULTS**

**Table A5: Hospitalisation costs in the year of index hip fracture (HES data April 2009 - March 2013)**

|  | **FCE-level reference costs** | | **Spell-level reference costs** | | **Spell-level tariffs** | |
| --- | --- | --- | --- | --- | --- | --- |
| Patient-years | 8,598 | | 8,598 | | 8,598 | |
| Number of observations | 11,184 | | 11,184 | | 11,184 | |
| Number of patients | 11,184 | | 11,184 | | 11,184 | |
| Distributional form | Gamma | | Gamma | | Gamma | |
| Link function | Identity | | Identity | | Identity | |
| **Parameters** | **Mean** | **SE** | **Mean** | **SE** | **Mean** | **SE** |
| Constant | 13,368 | 203 | 11,697 | 156 | 10,009 | 147 |
| Death within 30 days of hip fracture | -3,801 | 181 | -1,871 | 151 | -2,838 | 137 |
| Death within year of hip fracture | 3,427 | 245 | 2,097 | 173 | 1,812 | 178 |
| Living in care home | 2,524 | 233 | 1,230 | 155 | 1,976 | 166 |
| Female | -1,325 | 200 | -1,187 | 157 | -922 | 147 |
| Major non hip fracture | 6,332 | 732 | 4,273 | 490 | 3,692 | 497 |
| Second hip fracture | 10,437 | 736 | 8,827 | 530 | 7,446 | 524 |

SE = standard error.

**Table A6: Hospitalisation costs in year of second hip fracture (subsequent years to index hip fracture) (HES data April 2009 - March 2013)**

|  | **FCE-level reference costs** | | **Spell-level reference costs** | | **Spell-level tariffs** | |
| --- | --- | --- | --- | --- | --- | --- |
| Patient-years | 592 | | 592 | | 592 | |
| Number of observations | 652 | | 652 | | 652 | |
| Number of patients | 652 | | 652 | | 652 | |
| Distributional form | Gamma | | Gamma | | Gamma | |
| Link function | Identity | | Identity | | Identity | |
| **Parameters** | **Mean** | **SE** | **Mean** | **SE** | **Mean** | **SE** |
| Constant | 12,947 | 386 | 19,893 | 3,689 | 17,842 | 3,832 |
| Death within 30 days of hip fracture | -2,857 | 839 | - | - | -1,902 | 672 |
| Death within year of hip fracture | 4,324 | 1,438 | 3,438 | 839 | 2,323 | 835 |
| Age at second hip fracture | - | - | -102 | 42 | -100 | 44 |
| Living in care home | - | - | - | - | 1,250 | 554 |

SE = standard error.

**Table A7: Hospitalisation costs in subsequent years of index hip fracture (conditional on hospital admission) (HES data April 2009 - March 2013)**

|  | **Probability of hospitalisation in the years post first hip fracture** | | **FCE-level reference costs** | | **Spell-level reference costs** | | **Spell-level tariffs** | |
| --- | --- | --- | --- | --- | --- | --- | --- | --- |
| Patient-years | 11,850 | | 4,187 | | 4,187 | | 4,187 | |
| Number of observations | 12,509 | | 4,563 | | 4,563 | | 4,563 | |
| Number of patients | 9,185 | | 4,014 | | 4,014 | | 4,014 | |
| Distributional form | Binomial | | Gamma | | Gamma | | Gamma | |
| Link function | Logit | | Identity | | Identity | | Identity | |
| **Parameters** | **Mean** | **SE** | **Mean** | **SE** | **Mean** | **SE** | **Mean** | **SE** |
| Constant | -0.450 | 0.043 | 10,088 | 1,773 | 9,244 | 969 | 8,776 | 1,517 |
| Current age | - | - | -47 | 21 | -52 | 11 | -46 | 18 |
| Female | -0.274 | 0.047 | -973 | 309 | -818 | 216 | -631 | 234 |
| Living in care home | 0.348 | 0.045 | 1,757 | 267 | 504 | 177 | 944 | 193 |
| History of second hip fracture | 0.333 | 0.101 | - | - | - | - | - | - |

SE = standard error.

**Table A8: Hospitalisation costs in year of major non hip fracture (subsequent years to index hip fracture) (HES data April 2009 - March 2013)**

|  | **FCE-level reference costs** | | **Spell-level reference costs** | | **Spell-level tariffs** | |
| --- | --- | --- | --- | --- | --- | --- |
| Patient-years | 522 | | 522 | | 522 | |
| Number of observations | 556 | | 556 | | 556 | |
| Number of patients | 528 | | 528 | | 528 | |
| Distributional form | Gamma | | Gamma | | Gamma | |
| Link function | Identity | | Identity | | Identity | |
| **Parameters** | **Mean** | **SE** | **Mean** | **SE** | **Mean** | **SE** |
| Constant | 11,893 | 1495 | 6,107 | 339 | 5,728 | 346 |
| Female | -3,353 | 1527 | - | - | - | - |
| Living in care home | 3,179 | 823 | 1,886 | 586 | 2,013 | 635 |

SE = standard error.

**Table A9: Hospitalisation costs if death occurs (conditional on hospitalisation) (HES data April 2009 - March 2013)**

|  | **Probability of hospitalisation given death** | | **FCE-level reference costs** | | **Spell-level reference costs** | | **Spell-level tariffs** | |
| --- | --- | --- | --- | --- | --- | --- | --- | --- |
| Patient-years | 2,614 | | 1,600 | | 1,600 | | 1,600 | |
| Number of observations | 5,619 | | 3,246 | | 3,246 | | 3,246 | |
| Number of patients | 5,619 | | 3,246 | | 3,246 | | 3,246 | |
| Distributional form | Binomial | | Gamma | | Gamma | | Gamma | |
| Link function | Logit | | Identity | | Identity | | Identity | |
| **Parameters** | **Mean** | **SE** | **Mean** | **SE** | **Mean** | **SE** | **Mean** | **SE** |
| Constant | 3.494 | 0.353 | 8,113 | 328 | 5,899 | 204 | 6,093 | 236 |
| Current age | -0.033 | 0.004 | - | - | - | - | - | - |
| Female | -0.235 | 0.067 | -1,456 | 350 | -905 | 231 | -1,109 | 249 |
| Living in care home | -0.251 | 0.055 | 846 | 286 | - | - | 533 | 199 |

SE = standard error.

**Table A10: Non-hospitalisation costs in the year of index hip fracture (HES data April 2009 - March 2013)**

|  | **Reference costs** | | **Tariffs** | |
| --- | --- | --- | --- | --- |
| Patient-years | 8,598 | | 8,598 | |
| Number of observations | 11,184 | | 11,184 | |
| Number of patients | 11,184 | | 11,184 | |
| Distributional form | Gamma | | Gamma | |
| Link function | Log | | Log | |
| **Parameters** | **Mean** | **SE** | **Mean** | **SE** |
| Constant | 8.222 | 0.099 | 8.376 | 0.117 |
| Death within 30 days of hip fracture | -1.223 | 0.033 | -1.536 | 0.038 |
| Death within year of hip fracture | -0.271 | 0.031 | -0.377 | 0.040 |
| Age at index hip fracture | -0.021 | 0.001 | -0.024 | 0.001 |
| Female | -0.100 | 0.026 | -0.125 | 0.033 |
| Major non hip fracture | 0.541 | 0.042 | 0.584 | 0.066 |
| Second hip fracture | 0.461 | 0.046 | 0.410 | 0.066 |

SE = standard error.

**Table A11: Non-hospitalisation costs in year of second hip fracture (subsequent years to index hip fracture) (HES data April 2009 - March 2013)**

|  | **Reference costs** | | **Tariffs** | |
| --- | --- | --- | --- | --- |
| Patient-years | 592 | | 592 | |
| Number of observations | 652 | | 652 | |
| Number of patients | 652 | | 652 | |
| Distributional form | Gamma | | Gamma | |
| Link function | Identity | | Identity | |
| **Parameters** | **Mean** | **SE** | **Mean** | **SE** |
| Constant | 704 | 32 | 577 | 32 |
| Death within 30 days of hip fracture | -265 | 60 | -253 | 53 |

SE = standard error.

**Table A12: Non-hospitalisation costs in subsequent years of index hip fracture (conditional on non-hospitalisation contact) (HES data April 2009 - March 2013)**

|  | **Probability of non-hospitalisation costs in the years post 1^st^ hip fracture** | | **Reference costs** | | **Tariffs** | |
| --- | --- | --- | --- | --- | --- | --- |
| Patient-years | 11,850 | | 4,187 | | 4,187 | |
| Number of observations | 12,509 | | 4,563 | | 4,563 | |
| Number of patients | 9,185 | | 4,014 | | 4,014 | |
| Distributional form | Binomial | | Gamma | | Gamma | |
| Link function | Logit | | Identity | | Identity | |
| **Parameters** | **Mean** | **SE** | **Mean** | **SE** | **Mean** | **SE** |
| Constant | 2.655 | 0.222 | 556 | 19 | 525 | 19 |
| Current age | -0.023 | 0.003 | - | - | - | - |
| Female | -0.176 | 0.051 | -56 | 21 | -57 | 22 |
| History of major non hip fracture | 0.324 | 0.134 | - | - | - | - |
| History of second hip fracture | 0.209 | 0.106 | - | - | - | - |

SE = standard error.

**Table A13: Non-hospitalisation costs in year of major non hip fracture (subsequent years to index hip fracture) (HES data April 2009-March 2013)**

|  | **Reference costs** | | **Tariffs** | |
| --- | --- | --- | --- | --- |
| Patient-years | 522 | | 522 | |
| Number of observations | 556 | | 556 | |
| Number of patients | 528 | | 528 | |
| Distributional form | Gamma | | Gamma | |
| Link function | Identity | | Identity | |
| **Parameters** | **Mean** | **SE** | **Mean** | **SE** |
| Constant | 793 | 44 | 719 | 47 |

SE = standard error.

**Table A14: Non-hospitalisation costs if death occurs (conditional on non-hospitalisation contact) (HES data April 2009-March 2013)**

|  | **Probability of non-hospitalisation costs given death** | | **Reference costs** | | **Tariffs** | |
| --- | --- | --- | --- | --- | --- | --- |
| Patient-years | 2,614 | | 1,600 | | 1,600 | |
| Number of observations | 5,619 | | 3,246 | | 3,246 | |
| Number of patients | 5,619 | | 3,246 | | 3,246 | |
| Distributional form | Binomial | | Gamma | | Gamma | |
| Link function | Logit | | Identity | | Identity | |
| **Parameters** | **Mean** | **SE** | **Mean** | **SE** | **Mean** | **SE** |
| Constant | 3.765 | 0.263 | 493 | 18 | 420 | 19 |
| Current age | -0.039 | 0.003 | - | - | - | - |
| Female | -0.276 | 0.051 | -78 | 20 | -76 | 21 |
| Living in care home | -0.197 | 0.043 | - | - | - | - |

SE = standard error.

**Table A15: Top five HRGs with frequency and cost by HRG-based set of unit costs (2014-15 price year)**

|  | **FCE-level reference cost** | | | | | | **Spell-level reference cost** | | | | | | **Spell-level tariffs** | | | | | |
| --- | --- | --- | --- | --- | --- | --- | --- | --- | --- | --- | --- | --- | --- | --- | --- | --- | --- | --- |
|  | HRG ^a^ | Freq. | Cost ^b^ | Trim point ^c^ | Excess bed day cost | Estimated cost^e^ | HRG ^a^ | Freq. | Cost ^d^ | Trim point ^c^ | Excess bed day cost | Estimated cost^e^ | HRG ^a^ | Freq. | Cost ^b^ | Trim point ^c^ | Excess bed day cost | Estimated cost^e^ |
| 1 | HT12E | 2,064 | £6,625 | 23 | £266 | £6,885 | HT12E | 2,061 | £7,409 | - | - | £7,404 | HA12C | 2,475 | £6,321 | 29 | £235 | £6,761 |
| 2 | HT13E | 1,727 | £5,650 | 76 | £305 | £6,083 | HT13E | 1,729 | £6,316 | - | - | £6,312 | HA12B | 1,725 | £8,414 | 52 | £235 | £8,892 |
| 3 | HT12D | 1,382 | £7,487 | 31 | £292 | £7,840 | HT12D | 1,386 | £8,832 | - | - | £8,790 | HA13C | 1,451 | £5,631 | 29 | £235 | £6,138 |
| 4 | HT13D | 1,121 | £6,815 | 31 | £287 | £7,303 | HT13D | 1,123 | £8,160 | - | - | £8,141 | HA14C | 831 | £1,513 | 24 | £235 | £2,145 |
| 5 | HT12C | 575 | £8,909 | 44 | £260 | £9,289 | HT12C | 576 | £10,497 | - | - | £10,459 | HA13A | 797 | £7,017 | 60 | £235 | £7,422 |

^a^ See **Table A16** for definitions of the HRGs. ^b^ Non-elective long stay cost excludes excess bed days. ^c^ Bed days outside nationally set lengths of stay that are valued as excess bed day costs. ^d^ Non-elective long stay cost includes excess bed days. ^e^ includes long-stay and one day non elective admissions. Freq. = Frequency.

**Table A16: Definitions of the HRGs in Table A15**

|  | **HRG4+** | **Freq.** | **Definition** | **HRG4** | **Freq.** | **Definition** |
| --- | --- | --- | --- | --- | --- | --- |
| 1 | HT12E | 2,064 | Very Major Hip Procedures for Trauma with CC Score 0-2 | HA12C | 2,475 | Major Hip Procedures category 1 for Trauma without CC |
| 2 | HT13E | 1,727 | Major Hip Procedures for Trauma with CC 0-2 | HA12B | 1,725 | Major Hip Procedures category 1 for Trauma with CC |
| 3 | HT12D | 1,382 | Very Major Hip Procedures for Trauma with CC Score 3-5 | HA13C | 1,451 | Intermediate Hip Procedures for Trauma without CC |
| 4 | HT13D | 1,121 | Major Hip Procedures for Trauma with CC 3-5 | HA14C | 831 | Minor Hip Procedures for Trauma without CC |
| 5 | HT12C | 575 | Very Major Hip Procedures for Trauma with CC Score 6-8 | HA13A | 797 | Intermediate Hip Procedures for Trauma with Major CC |

**Table A15** reports the top 5 HRGs in our dataset for index admissions with one FCE (n=9,257) according to the three sets of unit costs. Spell-level reference costs were higher than FCE-level reference costs as they include excess bed days whereas the latter do not. Spell-level tariffs use HRG4 codes rather than the HRG4+ codes that inform the reference costs. For example, the most frequent HRG using the HRG4+ system is *‘Very Major Hip Procedures for Trauma with Complication Score 0-2*’ (HRG4+: HT12E) as opposed to ‘*Major Hip Procedures Category 1 for Trauma without Complications*’ (HRG4: HA12C) when using HRG4.

**Figure A3: Cost-effectiveness acceptability curves, by source of unit cost**

**REFERENCES**

1. Leal J, Gray AM, Hawley S, Prieto-Alhambra D, Delmestri A, Arden NK, et al. Cost-Effectiveness of Orthogeriatric and Fracture Liaison Service Models of Care for Hip Fracture Patients: A Population-Based Study. J Bone Miner Res. 2017;32(2):203-11.

2. Curtis LB, A. Unit Costs of Health and Social Care 2016. Canterbury: Personal Social Services Research Unit, University of Kent; 2016.

3. Briggs AS, M. Claxton, K. Decision Modelling for Health Economic Evaluation: Oxford University Press; 2006.

4. Hawley S, Javaid MK, Prieto-Alhambra D, Lippett J, Sheard S, Arden NK, et al. Clinical effectiveness of orthogeriatric and fracture liaison service models of care for hip fracture patients: population-based longitudinal study. Age Ageing. 2016;45(2):236-42.

5. HM Treasury. The Green Book: appraisal and evaluation in central government. <https://wwwgovuk/government/publications/the-green-book-appraisal-and-evaluation-in-central-governent2013>. Accessed 28 Jan 2018.
